# A pilot exploratory study examining the potential influence of continuous positive airway pressure devices on cranial molding trajectories in preterm infants

**DOI:** 10.1371/journal.pone.0292671

**Published:** 2023-10-12

**Authors:** Dana B. McCarty, Ashley Hite, Anna Brown, Kerry Blazek, Lauren Quinn, Sara Hammond, Marcella Boynton, T. Michael O’Shea

**Affiliations:** 1 Division of Physical Therapy, Department of Health Sciences, University of North Carolina at Chapel Hill School of Medicine, Chapel Hill, North Carolina, United States of America; 2 Department of Rehabilitation Services, Atrium Health Wake Forest Baptist, Winston-Salem, North Carolina, United States of America; 3 Department of Rehabilitation Services, UNC Children’s Hospital, Chapel Hill, North Carolina, United States of America; 4 Division of General Medicine and Clinical Epidemiology, Department of Medicine, University of North Carolina at Chapel Hill School of Medicine, Chapel Hill, North Carolina, United States of America; 5 Division of Neonatal-Perinatal Medicine, Department of Pediatrics, University of North Carolina at Chapel Hill, Chapel Hill, North Carolina, United States of America; Kobe University Graduate School of Medicine School of Medicine, JAPAN

## Abstract

**Objective:**

The objective of this exploratory study was to assess the potential impacts of two different continuous positive airway pressure (CPAP) devices on preterm infant head shape and circumference.

**Study design:**

Twenty infants born at <32 weeks gestational age requiring CPAP support were enrolled. Ten infants used the Hudson RCI Nasal Prong CPAP device and 10 infants used the Fisher-Paykel CPAP device. Infant Cranial Index (CI) and head circumference (HC) were collected weekly as well as infant gestational age at birth, and total number of days on CPAP.

**Results:**

At baseline, average total birthweight of infants was 1021 grams (SD = 227 grams), average gestational age was 26.9 weeks (SD = 1.80), mean CI was 79.7 cm (SD = 5.95), and HC was 10.2 cm (SD = 0.92). Days on CPAP ranged from 16 to 63 days, with an average of 40.7 (SD = 13.6) days. Neither CI nor HC differed by device type; however, the Fisher-Paykel device was associated with slightly greater HC growth rate.

**Conclusion:**

CPAP devices and the pressures they apply plausibly contribute to preterm infant cranial molding over time, with the greatest potential impact on infants who require CPAP support for longer periods; however, these findings must be validated in larger cohorts. Additionally, positioning practices should be further examined to determine how they may contribute to or prevent the development of cranial molding deformity.

## Introduction

Infant bones, especially those of preterm infants, have a high rate of collagen production, greatly increasing malleability and susceptibility to deformation [1]. Dolichocephaly, also known as positional scaphocephaly, is defined as a boat-shaped or elongated anteroposterior axis of the head [2]. This cranial molding deformity typically occurs in preterm infants as a result of frequent side-to-side head positioning of the infant during hospitalization [1, 2] and has reported prevalence ranges from 25–82% in hospitalized preterm infants [2, 3]. The high prevalence of cranial molding deformity reflects the ubiquity of sidelying or prone positioning of infants to improve respiratory mechanics [4], reduce reflux episodes, and prevent apneic and bradycardic events [2, 5, 6]. This deformity, however, can lead to multiple developmental concerns, including the secondary development of plagiocephaly and torticollis, motor asymmetries, delayed reaching skills, decreased midline control, myopia, and shifts in cortical structures in the brain [7–9]. Our previous research indicates that infants with dolichocephaly at 32 to 34 weeks postmenstrual age (PMA) are more likely than infants without this condition to be referred for physical therapy and treatment at 3–4 postnatal months [10].

Due to the underdevelopment of lung structures and absence of surfactant, nearly all infants born at <32 weeks gestational age have pulmonary insufficiency [11] and are at risk for developing respiratory distress syndrome [4, 11]. Continuous Positive Airway Pressure (CPAP) provides continuous airflow and blood gas stabilization and is often initiated soon after birth in cases of prematurity [11] to improve lung compliance and function [6]. CPAP devices are non-invasive and deliver positive pressure through nasal prongs or a nasal mask secured to the infant’s face and head [6, 11]. Non-invasive ventilation interventions like CPAP have been used increasingly in NICUs over recent years as an alternative to endotracheal intubation and mechanical ventilation [12]. This practice has reduced the incidence of adverse effects associated with more invasive forms ventilation (e.g., barotrauma to lungs) [12]. Depending on the model and design of a CPAP device, a variety of attachment points using Velcro straps, clips, and infant caps are used to securely position the CPAP to maintain a seal for optimized airflow. Because these devices apply pressure to the infant cranium and potentially restrict how the infant can be positioned, certain designs may perpetuate or exacerbate abnormal cranial molding [10, 13, 14].

Physical therapists (PTs) and occupational therapists (OTs) play an important role on a preterm infant’s multidisciplinary medical team by providing positioning recommendations [15] and monitoring infant musculoskeletal deformity [7] in the NICU setting. Consequently, it is incumbent upon these therapists to understand the potential implications of life-saving medical treatments on the infant musculoskeletal system. Because preterm infants are at increased risk for developing cranial molding deformities [2, 16], therapists and caregivers must consider any potential environmental contributors that could exacerbate cranial deformity risk. To our knowledge, no published study, to date, has examined the potential impact of CPAP device design on cranial molding in preterm infants. The objective of this study was to assess the potential effects of two different CPAP devices on infant head shape and circumference over the course of CPAP use. Based on the design of the two CPAP devices, subjective observations, and positions that each device accommodated, we hypothesized that infants wearing the Hudson prong CPAP would have larger head circumference and that infants wearing the Fisher-Paykel CPAP would demonstrate more elongated head shapes as indicated by the cranial index measure.

## Materials and methods

### Study design

In July of 2019, an observational cohort study was approved by the University of North Carolina (UNC) Newborn Critical Care Center Quality Improvement Council. This study was determined to be “exempt” from full review by the UNC-Chapel Hill Institutional Review Board and granted a waiver of the requirement for informed consent because outcome measures used are part of standard of care therapy and nursing practice and did not present undue or greater burden to infants. Strengthening and Reporting of OBservational studies in Epidemiology (STROBE) [17] guidelines were followed during the development and writing of this manuscript.

### Participants and setting

A total of 20 infants receiving care in the UNC Newborn Critical Care Center were enrolled into this observational study. Once an IRB waiver was received, we began weekly measures of eligible infants, recruited in sequence with the therapy plan of care and meeting the following inclusion criteria: born <32 weeks gestational age, required CPAP support, and had active physical and occupational therapy orders. All infants who met inclusion criteria were included in the study with no omissions of any eligible infant. The unit planned to change CPAP devices in September of 2019; therefore, the first 10 infants enrolled to the study prior to this date received respiratory intervention using the Hudson RCI Nasal Prong CPAP device (Teleflex, Morrisville, NC) [18, 19]. After September 2019 when new devices were being used, the next 10 infants meeting inclusion criteria used the Fisher-Paykel CPAP device (Fisher & Paykel Healthcare Limited, Auckland, NZ and were enrolled [20, 21]. No power calculations were calculated to determine appropriate sample size based on time constraints that afforded only 10 infants eligible for head measures prior to CPAP device change. Both CPAP devices in this study demonstrate equivalent effectiveness for maintaining optimal pressures and saturations in the preterm infant population [22]. The parent of the infant pictured in this manuscript has given written informed consent for the photos to be published under CC BY 4.0.

### Description of CPAP devices

#### Hudson RCI nasal prong CPAP device

**Fig 1:**
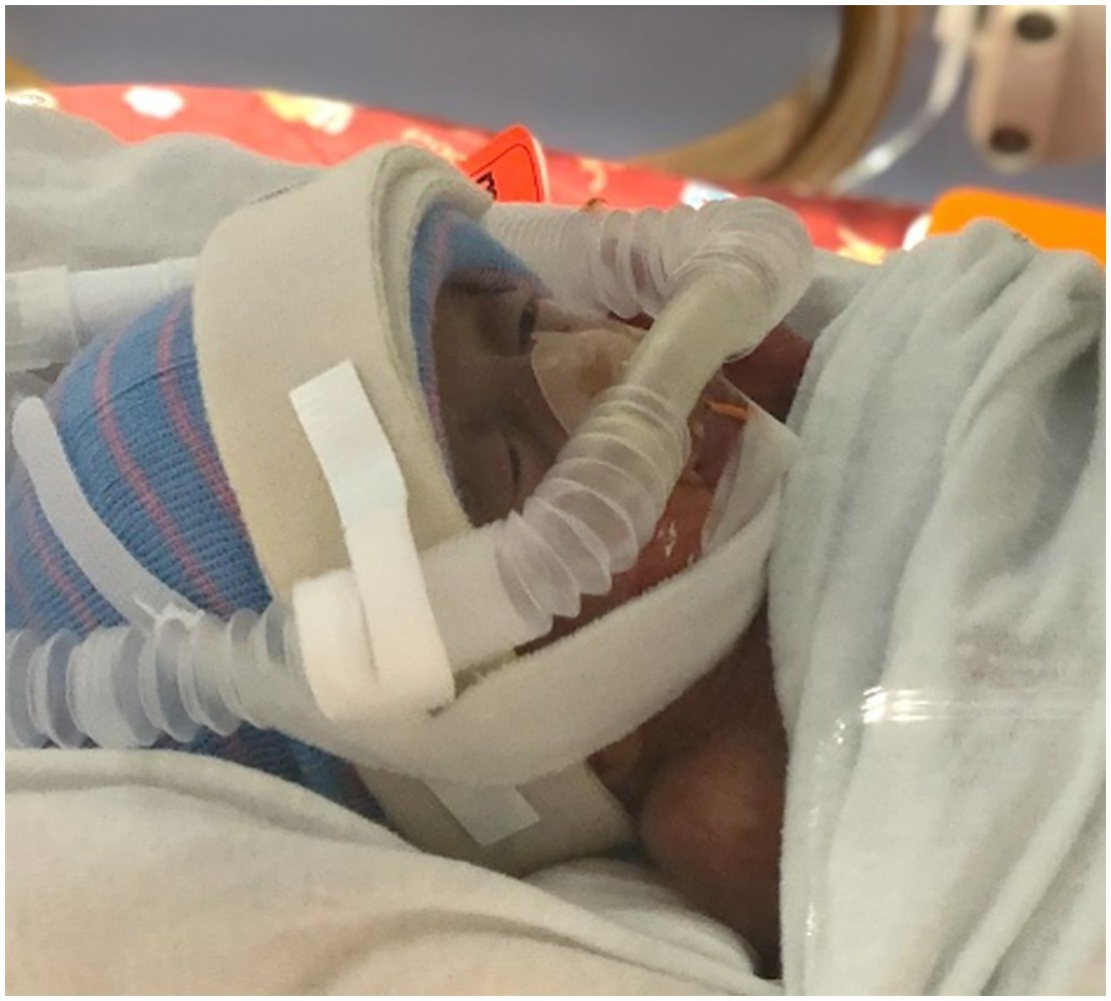
Hudson prong continuous positive airway pressure interface.

The Hudson device uses soft, curved prongs to reduce the risk of nasal septum breakdown and features adjustable right-angle connectors, or elbows, on each side of the infant’s face. The elbows connect the nasal prongs to two corrugated tubes that deliver continuous air pressure [18]. The tubing and nasal prongs are secured to the infant’s head and face using Velcro straps and a knit cap (Fig 1) [18]. The right-angle connectors are positioned such that prone and sidelying positions are difficult to achieve without causing the corrugated tubing to press into the infant’s cheek; therefore, infant positioning is most comfortably achieved using this device in supine or semi-sidelying position.

#### The Fisher-Paykel Bubble CPAP system

**Fig 2:**
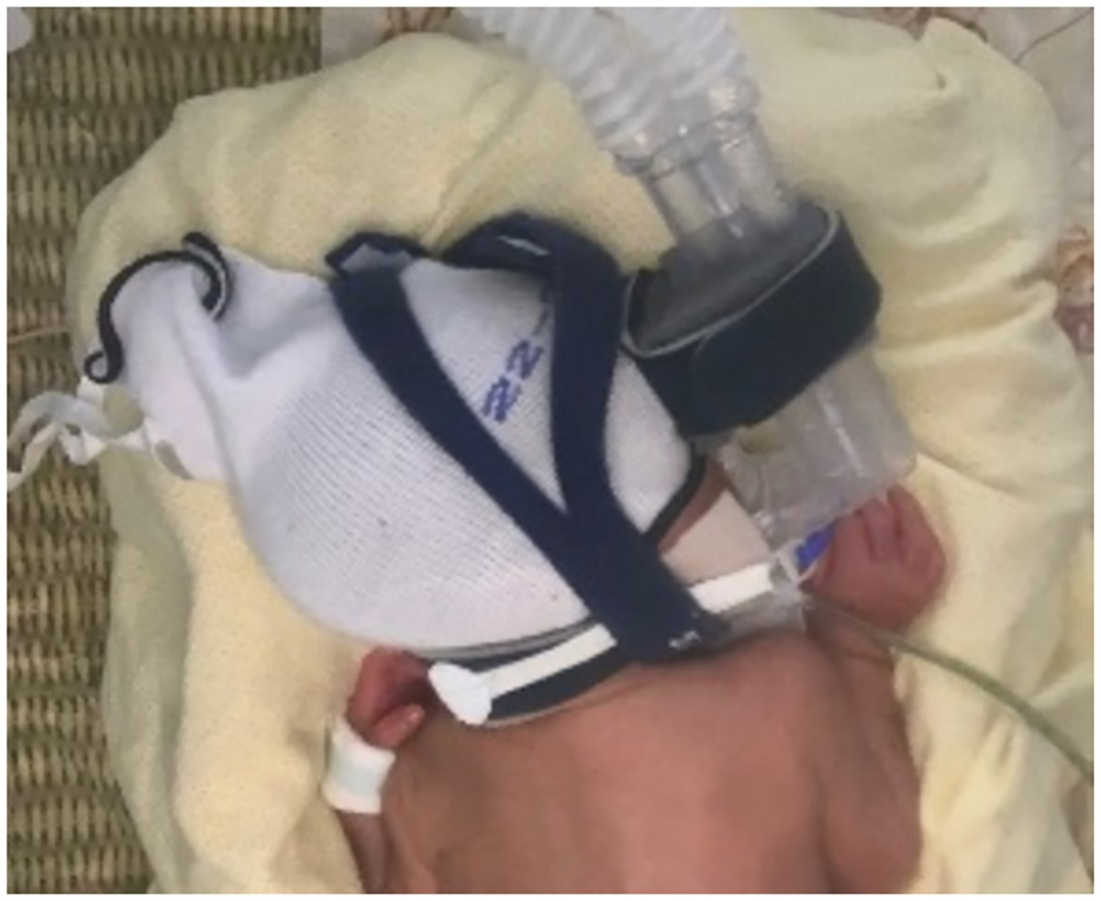
Fisher-Paykel continuous positive airway pressure interface. A dolichocephalic head shape can be observed as the infant lies prone with head in full right rotation.

The Fisher-Paykel (FP) Bubble CPAP system uses a “Flexitrunk Interface,” which is made up of nasal prongs and a nasal mask, a bonnet, and headgear to hold corrugated tubing in place [20, 21]. Unlike the Hudson device, in which two corrugated tubes are positioned along both sides of the infant’s face, the FP device situates two corrugated tubes in the midline. This design was created to offer more variability in infant positioning, including prone and sidelying. The device is secured to the infant’s face and head using Velcro straps and plastic clips that connect the nasal prongs or face mask to the infant’s bonnet or headgear (straps with Velcro) [20]. The nurse or respiratory therapist can choose to use a chin strap to keep the mouth closed and prevent air leaks (Fig 2) [20].

### Description of positioning and developmental care practices

In the Newborn Critical Care Center, nursing staff are trained to change the position of the infant every 3–4 hours at care times to support respiratory function, provide pressure relief, and to maintain midline flexion. Positions vary from supine, sidelying, semi-sidelying, and prone with head to the left or right. Multiple positioning aids were available to the nursing staff to assist with midline positioning and nesting of the infant, including fluidized positioning mattresses and pillows, prone pillows, bean bags, blanket swaddles, and blanket rolls. Each nurse used these positioning aids at their discretion and was expected to adjust positions based on infant tolerance. The nurses did not consistently document positioning changes in the electronic medical chart; therefore, infant position could not be included in the current analysis.

### Data collection

#### Measures

Infants were measured weekly from the time that CPAP use was initiated until CPAP was discharged, ranging from a minimum of 2 weeks to a maximum of 6 weeks. Infant Cranial Index (CI), which is also commonly referred to as the Cranial Proportional Index or Cephalic Index,(13) was measured weekly by the infant’s PT or OT from the time of CPAP initiation. The cranial index is measured using cranial calipers once the infant’s hat is removed and calculated by dividing the width of the infant’s head by the length in centimeters, and then multiplying by 100 to be reported as a percentage [13]. This method of measurement has high intra-rater reliability [3, 23] and demonstrates high correlation (r = 0.79–0.98) with 3-dimensional scanning technology [24]. In published studies, dolichocephaly has been defined as a CI of 76% or less [2, 13]. Prior to study initiation, in order to establish head measures in standard of care, 1 PT and 2 OTs were trained in caliper use by an experienced PT (DM) and achieved a sufficient level of inter-rater reliability by obtaining the same CI measures on a minimum of 5 infants each. Head circumference (HC), measured in centimeters, was collected by bedside nurses weekly and was recorded in the electronic medical record. Head circumference was determined by gradually moving the tape measure to obtain the greatest occipto-frontal diameter [25]. Cranial Index and head circumference measures were selected because of their common use and availability to our therapy and nursing teams for use in the NICU. Data collected from the electronic medical record included weekly head circumference documented by the nurse, infant birthweight in grams, infant gestational age at birth, total number of days on CPAP, and presence of intraventricular hemorrhage (IVH) of grade III or IV. All infant medical charts were screened for diagnosis of osteopenia of prematurity and low alkaline phosphatase levels as indicators of bone health, but no infants in our study had these characteristics.

### Statistical analysis

We tested a set of two linear multilevel models (MLM), one each for the outcomes of CI and HC, controlling for infant gestational age and birthweight. Because these data were fairly normally distributed, as evidenced by the similar mean and median values for CI and HC in Table 1, we used a linear mixed modelling approach. We conducted the analyses using maximum likelihood estimation, with the intercept and linear time treated as random and two-tailed critical alpha 0.05. To aid in model estimation and interpretation, infant birthweight in grams was grand mean centered, that is, the overall mean weight across time points was subtracted from each score. Text and tables report findings as unadjusted and adjusted unstandardized regression coefficients with adjusted coefficients are reported in the text.

**Table 1:** Infant characteristics and outcomes.

	Total	Hudson Prongs	Fisher-Paykel
	*M* ± SD or median, IQR	*M* ± SD or median, IQR	*M* ± SD or median, IQR
**Infant variable (Level 2)**	*N* = 20 patients	*n* = 10 patients	*n* = 10 patients
**Gestational age (weeks)**	26.9 ± 1.80	26.7 ± 1.39	27.7 ± 2.06
**Birthweight in grams**	1021.0 ± 228.7	957.5 ± 179.3	1084.4 ± 263.4
**Total days on CPAP**	40.7 ± 13.6	42.1 ± 13.8	39.3 ± 14.1
**Presence of Grade III/IV IVH**	n = 3	n = 2	n = 1
**Time-varying variable (Level 1)**	*n* = 68 observations	*n* = 38 observations	*n* = 30 observations
**Cranial Index (%)**			
**M ± SD**	79.7 ± 5.95	79.8 ± 5.57	79.6 ± 6.50
**Median, IQR**	80.0, 6	80.0, 7	80.0, 6
**Head circumference (cm)**			
**M ± SD**	24.7, 2.08	24.3 ± 1.97	25.0 ± 2.23
**Median, IQR**	25.0, 2.60	24.3, 2.40	25.0, 2.23

Note. M = mean; SD = standard deviation; IQR = interquartile range; CPAP = continuous positive airway pressure; IVH = intraventricular hemorrhage

## Results

At baseline, average total birthweight of infants was 1021.0 grams (SD = 228.7 grams), average gestational age was 26.9 weeks (SD = 1.80), mean CI was 79.7 cm (SD = 5.95), and HC was 10.2 cm (SD = 0.92). Days on CPAP ranged from 16 to 63 days, with an average of 40.7 (SD = 13.6) days. Two infants in Hudson Prongs group had diagnosed IVH Grade III or IV, and one infant in the Fisher-Paykel had diagnosed IVH Grade III or IV (Table 1).

**Fig 3:**
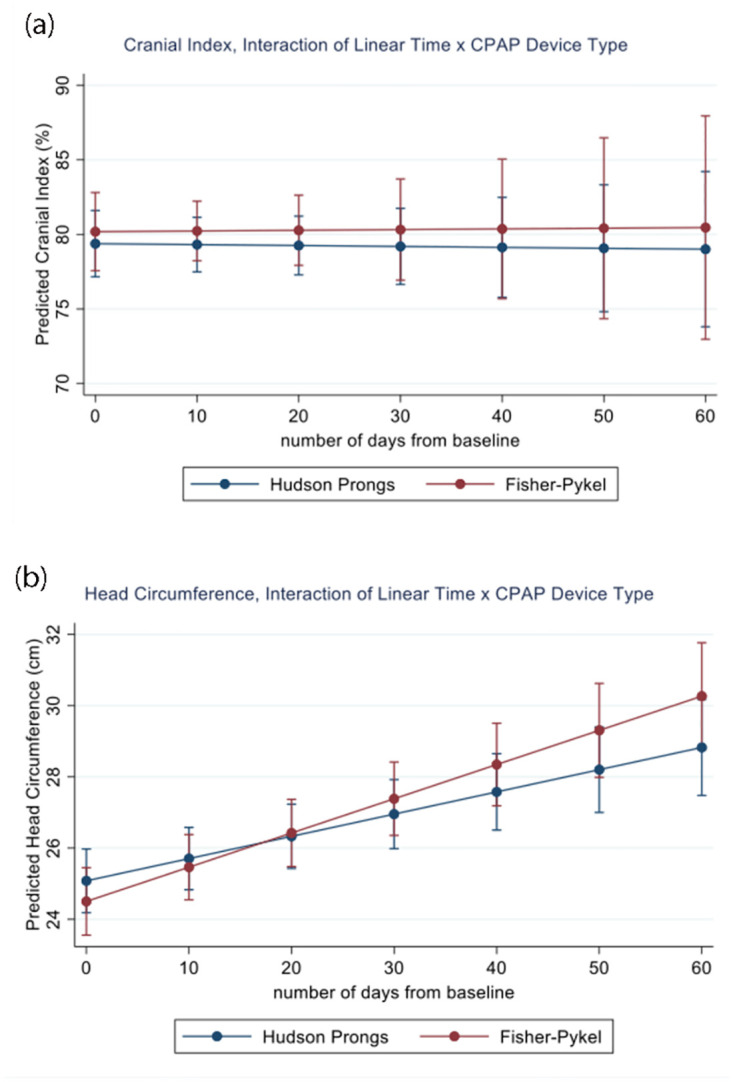
**a.** Estimated Cranial Index by Number of Days from Baseline Measurement. **b.** Estimated Head Circumference by Number of Days from Baseline Measurement.

Table 2 provides all unadjusted and adjusted MLM estimates with associated 95% confidence intervals. For the CI MLM, the intraclass correlation (ICC) was .275, indicating that roughly 72% of the variability in CI was explained by time level effects and approximately 28% of the variability in CI was explained by infant level differences. We did not observe a statistically significant main effect for CI by device type, infant GA at birth, nor linear time. There was an effect for infant birthweight in both groups such that for every 1 gram increase in birthweight, there was an associated average decrease of -0.014 in CI (b = -0.014, p = .01). A non-significant interaction between time × device type was suggestive on a minimal to no effect of CPAP on CI (Fig 3a).

**Table 2:** Linear multilevel regression model (MLM) unadjusted and adjusted model estimates.

	Cranial Index				Head Circumference			
	unadjusted		adjusted		unadjusted		adjusted	
	b	95% CI	b	95% CI	b	95% CI	b	95% CI
Intercept	79.54**	77.70, 81.38	76.38**	40.17, 112.58	25.95**	25.13, 26.77	29.28**	12.50, 46.05
***Level 2 –Infant-level variable***								
CPAP Device	-0.21	-3.90, 3.47	0.81	-2.70, 4.32	0.42	-1.21, 2.05	-0.58	-1.90, 0.74
*Fisher-Paykel = 1 Hudson Prongs = 0*								
Gestational age	-1.22*	-2.10, -0.34	0.11	-1.23, 1.45	0.35	-0.09, 0.79	-0.16	-0.78, 0.47
Gestational weight in grams, grand mean centered	-.013**	-.019, -.0065	-0.014*	-0.02, -0.003	0.005	0.002, 0.008	0.007*	0.002, 0.01
***Level 1 –Time-level variable***								
Days on CPAP								
Linear time effect	-.0002	-.08, .08	-0.006	-0.11, 0.10	0.08**	0.06, 0.10	0.03**	0.02, 0.03
***Interaction***								
Linear time effect x CPAP	0.08	-0.09, 0.25	0.01	-0.17, .19	0.03**	0.003, 0.063	0.03*	0.002, .06
Intraclass correlation (ICC)	.275				.501			

Note:

** < .001,

* < .05;

CI = Confidence Interval; CPAP = continuous positive airway pressure

For the HC MLM, the ICC was .501, indicating that approximately half of the variability for HC was explained by time level effects and the remaining half explained by infant level differences. No main effect was observed for HC by device type, infant GA at birth, nor linear time. There was a main effect for infant birthweight; for every 1 gram increase in birthweight, there was an associated average increase of 0.007 in HC in centimeters (b = 0.007, p = .006). Notably, there was also a significant linear time × device interaction for HC such that, with increasing time on the CPAP device increases, head circumference also increases and at a faster rate for the Fisher-Paykel device (Fig 3b).

## Discussion

Our exploratory project revealed that the types of CPAP devices and the pressures they apply may have an impact on preterm infant cranial molding over time. The ICCs observed for CI and HC indicate significant dynamics in skull size and shape over time for the infants in this study. With this fact in mind, it is incumbent on those providing care to preterm infants to adopt treatment tools and care environments that maximally support optimal head shape development.

Lower birthweight and gestational age have been linked to worse cranial molding outcomes [2, 26], particularly for dolichocephaly. Concordant with this previous research, we found that higher gestational age at birth was associated with a larger HC, which indicates a rounder or larger head shape. However, we also found an association between larger birthweight and CI in the opposite direction such that infants in our sample with higher birthweights were more likely to demonstrate a lower CI, or narrowing, of the skull, regardless of device used. We anticipate that the association between greater birthweight and worsening CI may be attributable the fact that larger infant bodies can be more challenging to position securely in midline, especially when supine [4]. Stated differently, heavier infants may succumb to the effects of gravity to a greater extent, causing the extremities or trunk to move out of the midline and putting greater pressure on the skull. This difference in positioning can, due to gravitational pull and elicitation of postural reflexes, cause the head to rotate out of midline and reinforce asymmetric positioning [27]. Therefore, it is possible that larger infants may experience longer durations of time in asymmetric head positions, leading to lower CIs. Further examination of how infant size and age contribute to head shape in the NICU is needed.

**Fig 4:**
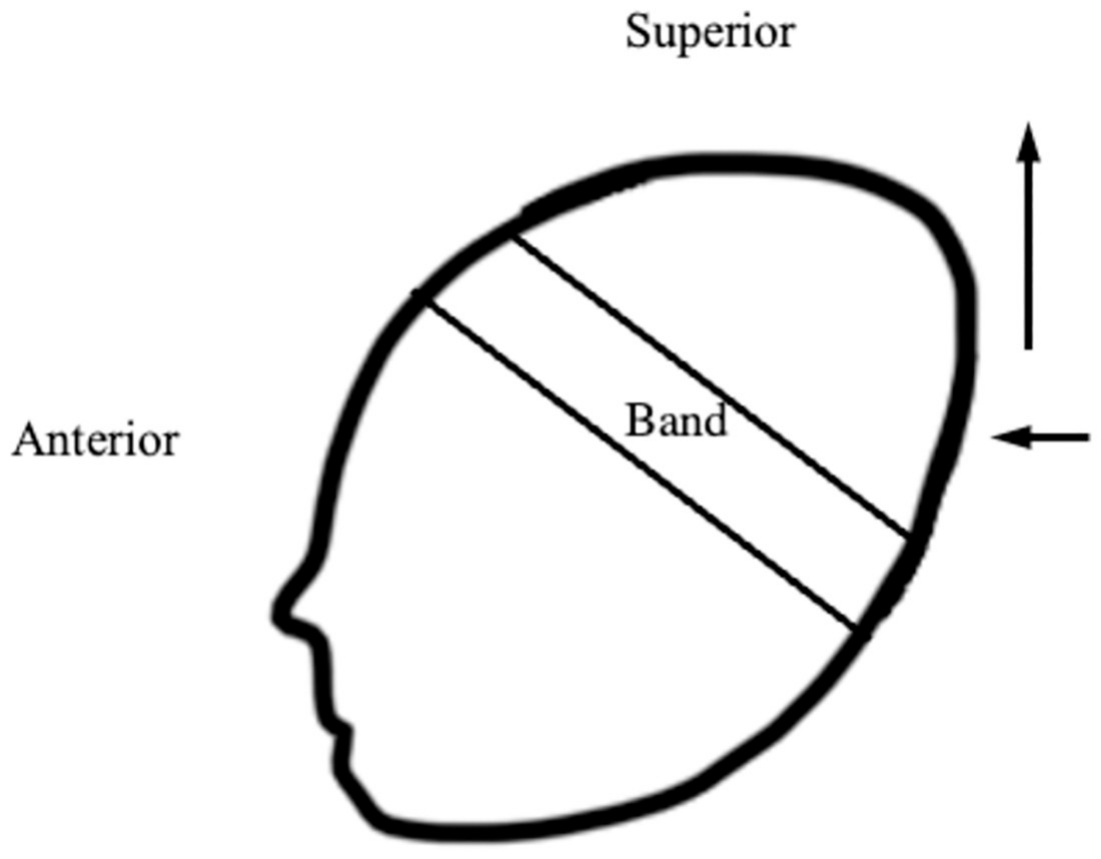
Hypothesized head shape of infants wearing Hudson CPAP device. The band illustrates the position of the fabric band that supplies attachment points for the CPAP interface. The left-pointing arrow indicates pressure created from the support surface of the mattress leading to a flattened posterolateral occiput from supine/semi-supine positioning. The upward-pointing arrow indicates superior protrusion of skull as the infant’s head grows in response to pressure from the circular band and posterolateral pressures from the support surface.

Based on HC and CI model results, we conclude that infants on Hudson prongs and FP CPAP devices tend to develop slightly different head shapes. Infant head shape characteristics for infants using Hudson prongs consisted of posterior symmetric flattening of the postero-lateral skull with resultant superior cranial protrusion (Fig 4). Based on our clinical observations, we believe that infants wearing the Hudson prong device may be subjected to greater pressure on the postero-lateral cranium due to device design contributing to frequent supine and semi-sidelying positioning. As previously mentioned, Hudson prongs do not allow full sidelying or prone due to widely positioned right angle connectors that prevent weightbearing on the side of the face. This positioning, along with the circumferential band affixing the interface, may create pressure in the postero-lateral skull so that as the head grows, it meets less resistance superiorly, resulting in disproportionate head growth upwards. This infant head shape may contribute to what appears to be slower rate of HC growth projections because the traditional landmarks for occipto-frontal HC measures [28] are affected by the posterior symmetric flattening of the occiput and cranial bulk shifting upwards with growth (Fig 4). While based on HC measures in this study, the CPAP did seem to contribute to formation of the head shape described above, we do not anticipate this head shape would contribute to poor midline positioning or development of later asymmetric head and neck preferences.

Characteristics of infant head shape observed in infants using the FP device consisted of bilateral symmetric or asymmetric flattening of the lateral parietal and temporal bones with occipital bossing, otherwise known as dolichocephaly (Fig 2). The FP interface is secured with head gear that includes a bonnet with circumferential strap, Velcro straps that secure the nasal piece of the interface to the bonnet, and a chin strap [20]. The FP interface instruction sheet suggests using the least amount of pressure possible to maintain the airway seal to reduce pressure on the face and head. Due to the midline positioning of the interface, infants can easily be positioned in sidelying or prone, positions which aid in respiration, with free weightbearing on the sides of the head. Because infants using the FP device may have experienced greater tension on the cranium due to the apparatus design and may also have experienced prone and sidelying positioning to a greater extent, we anticipated that these infants would develop dolichocephaly at higher rates. We did not, however, see any statistically significant differences in CI between groups—this null finding may be, in part, the result of our relatively small sample size.

### Limitations

Although the study has multiple strengths, among them being a longitudinal design, collection of robust measures of infant head shape comparing two different CPAP devices, and accounting for multiple potential confounders (e.g., infant birthweight) in the statistical models, these are somewhat offset by certain study limitations. One limitation of the current study was that the shape and restrictions of the incubator and medical devices can contribute to measurement error. Fortunately, CI measured with spreading calipers has good sensitivity (93%), high accuracy (96%) [29]. Unlike 3-dimensional scanners [24] used in the outpatient setting, calipers are more economical for clinical use in the NICU setting to track progress over time [29]. All study staff responsible for head measurement were trained in caliper use and established reliability on a minimum of 5 infants, producing identical width and length measurements prior to implementing caliper measures as standard of care. This intensive training approach likely mitigated measurement error issues, although future research might benefit from measuring skull deformity on more than one plane.

A second limitation was that the current study collected observational data on a relatively small cohort of infants. We were only able to study a small number of infants due to time constraints imposed by a change to the CPAP device type at the study site just a few months prior to the scheduled study date in September of 2019. The small scope of the study limited the number and type of potential confounders that could be included in the analyses and limited our ability to draw valid conclusions. Future work in this area would benefit studying larger cohorts of infants and the inclusion of additional clinical and outcome measures (e.g., parenteral nutrition regimen, medication use, bone mineral density).

We did not follow the group of 20 study infants beyond their NICU stay because of high rate of hospital transfer and limited resources for extended study follow up outside of the regional area. Future studies would benefit from studying larger infant cohorts over longer periods of time both during and after CPAP use and assessing both symmetrical (dolichocephaly) and asymmetrical (plagiocephaly) forms of cranial molding deformity. Additional research examining the use of positional protocols to prevent and treat development of dolichocephaly in the context of CPAP use is needed [10, 30, 31]. Future work should also examine the potential association between larger infant size and possible increased risk of dolichocephaly in the presence of CPAP.

An additional limitation is that CI and HC measures were only considered during the time of CPAP use. Future studies should examine changes in head shape throughout hospitalization, at the time of discharge, and at outpatient follow-up. Our team’s work did not assess number of days on CPAP; however, other research in this domain has shown significant associations between CI at hospital discharge and CI at hospital follow-up as well as a significant association between presence of dolichocephaly at 32–34 weeks gestation and need for PT services at outpatient follow-up [13].

We were unable to systematically assess any differences between CPAP device groups with respect to their effects on infant positioning. The designs of the CPAP devices afforded different positioning options and weightbearing potential on the skull, and nurses had great latitude to select the position that best suited patient needs. Because the devices have obvious design differences, nurses were not blind to study condition. As previously noted, the design of the CPAP device likely facilitated use of certain positions. Specifically, nurses may have found prone and sidelying more easily attained using the Fisher-Paykel device and supine positioning more feasible with the Hudson prongs device; however, this conclusion is based on informal feedback from the nursing staff and observations from the study authors and not on systematic quantitative assessment. That is, nursing documentation of infant position throughout the shift was completed <50% of care times due to NICU workflow concerns, which precluded use of this variable in our analyses.

### Clinical implications

The scope of neonatal physical therapy and occupational therapy continues to evolve as medical advances progress. With increased use of CPAP devices in the NICU, therapists must observe and consider the potential long-term musculoskeletal impacts on infant head shape development. While prematurity is a well-understood risk factor for cranial molding deformity because of the increased malleability of the skull [1], sub-optimal positioning strategies [4], and early exposure to gravity [4, 7], less is known about the consequences of long-term non-invasive forms of respiratory support in preterm infants on resulting head shapes.

Objective measures of cranial molding deformity in the NICU affords the physical and occupational therapist an opportunity to accurately assess change over time and respond appropriately to mitigate the various environmental effects on suboptimal head shape development. Through collaboration with nursing staff and other care providers, therapists can develop a positioning plan of care with recommendations that may include alternating supine, prone, and sidelying [10, 13], increasing supine positioning while maintaining the neck and airway in neutral alignment [4, 13], limiting prone positioning [4], and using support rolls and positioning aids to optimize midline positioning of the head and neck [4, 5, 10, 32]. Because cranial molding deformity influences the development of cervical muscle strength and length [16], it is critical for physical and occupational therapists to identify infants at greatest risk and intervene early.

## Conclusions

CPAP devices and the pressures they apply to an infant’s skull plausibly contribute to preterm infant cranial molding over time, with the greatest potential impact on infants who require CPAP support for longer periods. Device type was associated with differences in head growth (i.e., HC) across the study period. Greater weight at birth was also associated with increased narrowing of CI in the context of CPAP use. These findings must be validated in larger cohorts over longer periods of time. Additionally, positioning practices should be further examined to determine how they may contribute to or prevent the development of cranial molding deformity.

## Supporting information

S1 Dataset (XLSX)
